# Effect of patient anxiety on image motion artefacts in CBCT

**DOI:** 10.1186/s12903-017-0367-4

**Published:** 2017-04-04

**Authors:** Elif Yıldızer Keriş

**Affiliations:** Çanakkale Dentistry Hospital, Department of Radiology, Kepez, Çanakkale Turkey

**Keywords:** Cone beam computed tomography, Motion artefacts, Anxiety

## Abstract

**Background:**

Artefacts in images related to patient movement decrease image quality, potentially necessitating re-scanning, which leads to an extra radiation dose for the patient. Thus, avoiding patient motion reduces patient exposure to radiation. The aim of this study was to analyse image motion artefacts (MAs) and how they are affected by patient anxiety during cone beam computed tomography (CBCT) examination.

**Methods:**

A total of 100 patients undergoing CBCT examination were investigated. The State Trait Anxiety Inventory (STAI-S and STAI-T) form was used to measure patient anxiety. Patient’s age, gender, dental anxiety score, diagnostic reason for CBCT examination, field of view (FOV), acquisition time, anatomical area, and presence of motion artefacts on images were recorded. Comparisons of the parameters were evaluated using Pearson’s correlation, the chi-square test, the Mann-Whitney U test, the Kruskal-Wallis test and t-tests. The significance level was set at 0.05.

**Results:**

The mean values of the scores for the total population were 37.2 for the STAI-S and 41.6 for the STAI-T. Women exhibited higher anxiety levels than men. The patients’ anxiety scores were significantly correlated with dental fear. The prevalence of patients showing motion artefacts was 6%. The mean age of patients with motion artefacts on their images (56.83) was higher than that of patients without (39.14). There was no relationship between motion artefact presence and patient gender, anxiety score, diagnostic reason for CBCT examination, FOV, acquisition time, or anatomical area. Patients showing motion artefacts on their images had higher STAI scores than those with no motion artefacts (non-significant).

**Conclusions:**

The population in this study experienced anxiety before CBCT scanning. Excessive anxiety did not clearly affect whether image motion artefacts were generated during CBCT examination, although a non-significant increase in STAI scores was noticed in patients with motion artefacts on their images.

## Background

Cone beam computed tomography (CBCT) has become widespread in dentistry because it produces three-dimensional images with high resolution of bony structures and because the radiation dose of CBCT is lower than that of computed tomography (CT) [1, 2]. However, CBCT has some disadvantages, such as motion artefacts (MAs).

MAs are a general problem in radiology because the primary principals of radiology are to reduce radiation exposure to patients and to ensure the best image quality. Patients are asked to remain still during CBCT examinations to prevent MAs. However, even though CBCT scans last only seconds (6–36 s), patients still move during scanning [3]. Artefacts in images resulting from patient movement decrease image quality and the diagnostic performance of CBCT. Re-scanning is needed if image quality is not sufficient for diagnosing and reporting, which leads to an extra radiation dose for the patient.

To overcome this problem, the causes of patient movement must be clearly identified. Studies investigating MAs in maxillofacial CBCT images and patient movement during CBCT scanning are limited. Previous studies have assessed MA incidence retrospectively [4–6] and examined the impact of movement on image quality using phantoms/dry skulls or in vitro [7–9], by observing patient movement on video recordings taken during CBCT examinations [10, 11]. It is important to assess patient characteristics because MAs are related to patients.

According to select magnetic resonance imaging (MRI)-based studies, MAs are more commonly observed for anxious patients than calm patients [12, 13]. Anxiety is a general term for several disorders that cause nervousness, fear, apprehension, and worrying. It is well known that a patient’s anxiety increases before diagnostic and therapeutic procedures [14]. Hence, for maxillofacial CBCT use in dentistry, consideration should be given to the possibility that dental procedures could also be a triggering factor for anxiety [15–17]. For this reason, evaluating patient anxiety before maxillofacial CBCT scanning could be useful to avoid patient movement and protect patients from extra radiation doses by avoiding the need to repeat imaging. There are no published studies about correlations between patient anxiety and MAs in oral and maxillofacial CBCT images. The aim of this study was to analyse patient anxiety before CBCT examination and to investigate the relationship between this anxiety and the presence of image MAs.

## Methods

A total of 100 patients (61 females and 39 males; age range, 18–75 years) referred for CBCT examination at the Radiology Department of Çanakkale Dentistry Hospital in Turkey participated in the study. Ethical approval and permission to undertake the study were given by the Ethics Committee at Çanakkale Onsekiz Mart University. All patients provided informed consent prior to entering the study.

### Patient characteristics

The criteria for inclusion in the study were that the patients could speak and understand Turkish, had the physical and mental ability to complete three questionnaires without assistance and had no movement diseases such as Parkinson’s disease. The patients filled in anxiety questionnaires before undergoing CBCT scanning. Patient age and sex were noted on the questionnaires.

The State Trait Anxiety Inventory (STAI) form published by Spielberger et al. [18] was used to measure patient anxiety. The validity and reliability of the STAI is high [18], and it has been frequently used in radiologic studies [12, 13, 19–22]. The STAI-S (STAI Form Y-1) measures state anxiety, and the STAI-T (STAI Form Y-2) measures trait anxiety [23]. State anxiety reflects fear, nervousness, discomfort, and temporarily induced arousal of the autonomic nervous system. Trait anxiety denotes a relatively enduring disposition that suffers from stress, worry, and discomfort [23]. Both the STAI-S and the STAI-T consist of 20 statements with scores ranging from 1 to 4 for each question. A score of 1 means ‘not at all’, while 4 indicates ‘very much.’ Some of the questions are designed to evaluate positive emotions, whereas others describe negative emotions. The questions are provided in Table 1. The scores for the answers are reversed for positive questions during scaling. The total score obtained from both the STAI-S (STAI Form Y-1) and STAI-T (STAI Form Y-2) forms ranges from 20 to 80. Higher scores refer to higher anxiety levels, while lower scores refer to lower anxiety levels [18].

**Table 1:** STAI tests

STAI-S (STAI Form Y-1)	STAI-T (STAI Form Y-2)
1. I feel calm	1. I feel pleasant
2. I feel secure	2. I feel nervous and restless
3. I am tense	3. I feel satisfied with myself
4. I am regretful	4. I wish I could be as happy as others seem to be
5. I feel at ease	5. I feel like a failure
6. I feel upset	6. I feel rested
7. I am presently worrying about possible misfortunes	7. I am “calm, cool, and collected”
8. I feel rested	8. I feel that difficulties are piling up so that I cannot overcome them
9. I feel anxious	9. I worry too much over something that really doesn’t matter
10. I feel comfortable	10. I am happy
11. I feel self-confident	11. I have disturbing thoughts
12. I feel nervous	12. I lack self-confidence
13. I am jittery	13. I feel secure
14. I feel “high strung”	14. I make decisions easily
15. I am relaxed	15. I feel inadequate
16. I feel content	16. I am content
17. I am worried	17. Some unimportant thought runs through my mind and bothers me
18. I feel over-excited and rattled	18. I experience disappointments so keenly that I can’t put them out of my mind
19. I feel joyful	19. I am a steady person
20. I feel pleasant	20. I get in a state of tension or turmoil as I think over my recent concerns and interests

Corah’s Dental Anxiety Scale (DAS) [24], which is commonly used to assess dental anxiety [25–28], was administered as a third questionnaire to measure patient dental anxiety. The scale consists of 4 questions, each with 5 answer alternatives. DAS scores range from 4 (no anxiety) to 20 (highly anxious).

The patients were examined to identify the reason for CBCT imaging, and these reasons were categorized into seven groups: dental pain, implant planning, suspicion of cyst/tumour, trauma, temporomandibular disorder (TMD), maxillary sinus pathologies and other asymptomatic reasons such as impacted teeth and bifid mandibular canal.

### CBCT examination

**Fig. 1:**
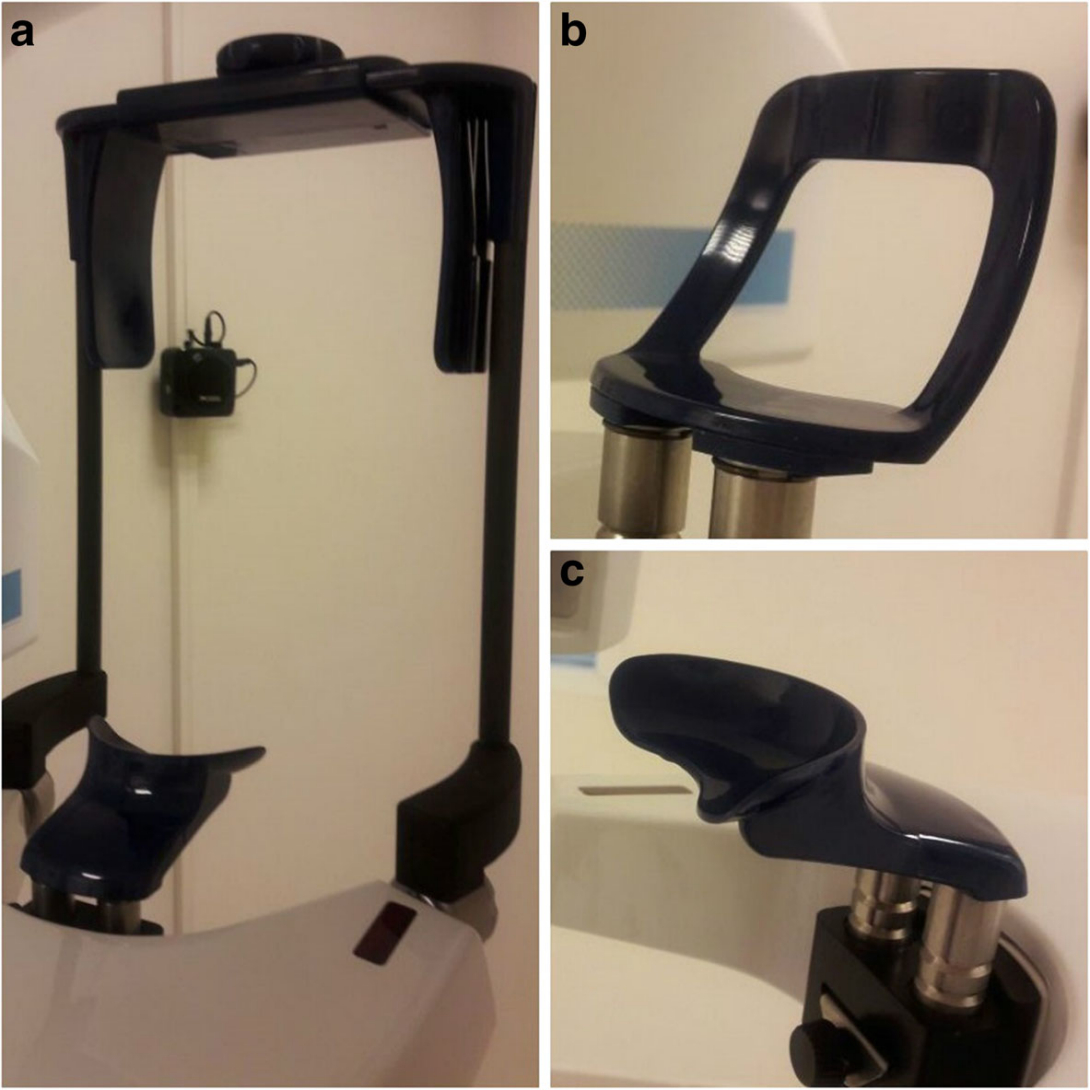
**a** Adjustable head support provided by the Planmeca Pro Max 3Ds unit. **b** Chin support. **c** Chin cup

CBCT examinations were performed using a ProMax 3Ds (Planmeca Oy, Helsinki, Finland). In this X-ray unit, patients can stand or sit during the exposure. The manufacturers suggest that patients should stand for ease of operation. According to the manufacturers’ suggestions, we guided all of the patients to stand during the study. The patient’s head was stabilized using two vertical support bars, an adjustable head support that slides onto the bars and a “chin support” or “chin cup” (Fig. 1). When taking 3-dimensional TMJ exposures, the patient was positioned on the chin support (pressing his/her lips against the chin support and closing his/her mouth while keeping the teeth together). When scanning the jaw, the patient was positioned on the chin cup (placing his/her chin on the chin cup while using a cotton roll to avoid contact between his/her upper and lower teeth). Patients were scanned with their eyes open.

The image acquisition parameters for the CBCT machine were 90 kVp, 6.3 mA, and 0.4 mm voxel size. The exposure values automatically changed according to patient size, image resolution and the “low dose” setting in the CBCT unit. We selected a “low dose” option for all of the patients; therefore, voxel sizes remained consistent for all acquisitions (based on the acquisition settings of the CBCT unit, the 0.4 mm voxel size was considered “low dose”).

The field of view (FOV) and acquisition time were changed according to the imaging protocol chosen by the CBCT operator. In this CBCT unit, all image volumes have the same diameter (5 cm). The volume height is selectable and can be 5 cm or 8 cm (one cylindrical volume can be 5 × 5 cm or 5 × 8 cm). According to the size of the target area, the FOV can be enlarged by the horizontal stitching of several volumes, and the image consists of two (double scan; *double* 5 × 5 cm or *double* 5 × 8 cm) or three (triple scan; *triple* 5 × 5 cm or *triple* 5 × 8 cm) cylindrical volumes. The volumes are automatically stitched by the software in the CBCT unit. In this study, the scan time ranged between 11.992 and 37.051 s, and the FOV sizes selected for the patients were 5 × 5 cm (13 patients), 5 × 8 cm (23 patients), *double* 5 × 5 cm (4 patients), *double* 5 × 8 cm (31 patients), *triple* 5 × 5 cm (12 patients), and *triple* 5 × 8 cm (17 patients).

Assessments of MAs were performed by a dentomaxillofacial radiologist in two separate sessions over at least two-week intervals. All images were displayed on a 19-in. LCD monitor (Dell Inc., Round Rock, TX, USA) in a dim room. Romexis Viewer (3.8.3.R, Planmeca Oy, Helsinki, Finland) software was used to evaluate the images in three orthogonal planes, and data on the image acquisition protocol were collected for each patient. During the evaluation of the images, anatomical regions of the scans were noted as jaws and temporomandibular joint (TMJ) areas because guidance of the patient to the CBCT unit differed for the two imaging modalities; this was in accordance with the recommendations in the Planmeca Promax3D s user’s manual (described above).

**Fig. 2:**
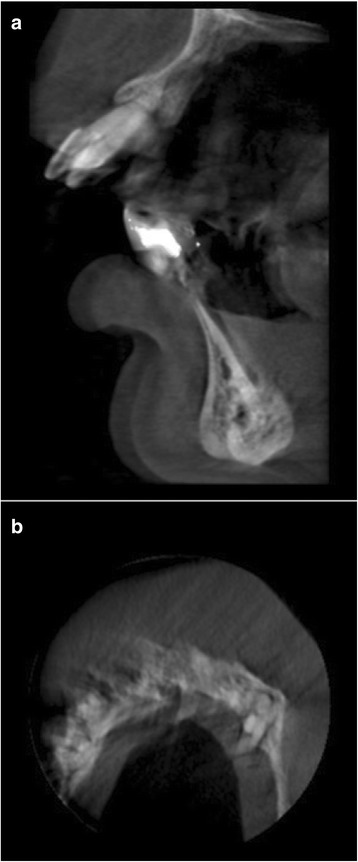
**a** Double bony contours due to motion of 36-year-old female patient. **b** Unsharpness of bony contours observed in axial scan

**Fig. 3:**
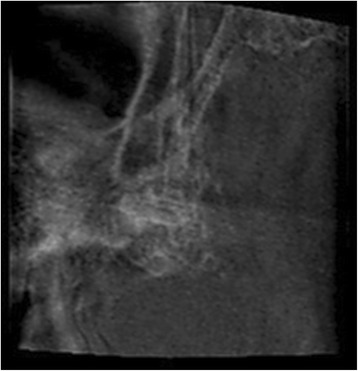
Motion artefact evident in CBCT image of the posterior region of the right jaw with 5 × 5 FOV and 12.01-s acquisition time

The observer was blinded to the patients’ characteristics during assessment of the images. A MA was recorded if “double or unsharpness of bony contours” were observed in the image, in agreement with previous studies [4, 9]. Examples of CBCT images showing signs of MAs are displayed in Figs. 2 and 3. If an image consisted of multiple volumes, each volume was first examined alone during the evaluation of image MAs because volumes can be incorrectly aligned during stitching. No incorrectly aligned volumes were found in the images.

Patient movements were not monitored during scanning because Turkish regulations state that patients cannot be monitored in clinical and radiographical examination areas in hospitals to protect patient privacy.

In addition, for patients with MAs, image quality was evaluated for diagnosis and reporting, and a requirement for re-scanning related to MAs was recorded.

### Statistical evaluation

Records were statistically analysed using SPSS (version 17.0; SPSS Inc., Chicago, IL). Kappa (κ) coefficients of intra-observer agreement for the evaluation of motion artefacts were calculated, and values of more than 0.7 were denoted as acceptable consistency. Relationship among anxiety scores and patient’s age were tested with Pearson’s correlation. Kruskal Wallis test was used to compare the anxiety scores and diagnostic reasons for CBCT imaging. We used independent samples t test to assess the correlations between anxiety scores and gender differences. Mann-Whitney U test was used for analysis of relations between motion artefacts and acquisition time/patient’s age/anxiety scores. Comparison between motion artefacts and FOV/anatomical area/gender differences/diagnostic reasons for CBCT examination was assessed by chi-square. Confidence intervals (CI) were estimated using bootstrap re-sampling. Differences were taken as significant at *P* < .05.

## Results

### Patient characteristics

Of the 100 patients, 61 were females and 39 were males, with a mean age of 40.2 years. The mean scores for the total population were 37.2 for the STAI-S, 41.6 for the STAI-T, and 8.5 for the DAS. Correlations among anxiety scores showed a significant positive relationship among STAI-S, STAI-T, and DAS values (*p* < 0.05, Pearson correlation). A non-significant negative relationship with a very low level (*p* > 0.05, *r* = −0.94, Pearson correlation) was found between STAI-S score and patient age, a non-significant positive relationship with a very low level (*p* > 0.05, *r* = 0.12, Pearson correlation) was found between STAI-T score and patient age, and a significant negative association was found between DAS score and patient age (*p* < 0.05, *r* = −0.209, Pearson correlation).

Correlations between anxiety score, gender and diagnostic reason for CBCT imaging are provided in Tables 2 and 3. Women had significantly higher STAI and DAS scores than men (*p* < 0.05, t test) (Table 2). No significant differences were found among anxiety scores for patients with different diagnostic reasons for CBCT (*p* > 0.05, Kruskal-Wallis) (Table 3).

**Table 2:** Correlations between anxiety score and gender

Gender x Anxiety score		*n*	Mean	SD	*p*
STAI-S	Female	61	39.10	10.56	0.022
	Male	39	34.31	9.23	
STAI-T	Female	61	43.38	8.59	0.011
	Male	39	38.92	8.06	
DAS	Female	61	9.36	3.68	0.001
	Male	39	7.13	2.64	

*n* sample size, *SD* standard deviation, *p* significance level, *STAI-S* State Trait Anxiety Inventory - State, *STAI-T* State Trait Anxiety Inventory - Trait, *DAS* Dental Anxiety Scale

**Table 3:** Relationships between anxiety score and diagnostic reason for CBCT imaging

	STAI-S* (mean, SD)	STAI-T^¥^ (mean, SD)	DAS^β^ (mean, SD)
Dental pain (*n* = 44)	39.77 (10.91)	42.25 (8.66)	8.84 (3.40)
Cyst/Tumour (*n* = 12)	39.83 (12.55)	43.25 (10.59)	9.42 (3.80)
TMD (*n* = 20)	35.30 (8.52)	39.55 (8.87)	8.15 (3.62)
Implant planning (*n* = 10)	31.70 (7.85)	39.80 (5.73)	7.10 (2.96)
Sinus pathologies (*n* = 4)	31.50 (10.25)	39.25 (10.66)	7.25 (1.71)
Trauma (*n* = 4)	33.75 (5.38)	43.75 (8.46)	7.75 (3.86)
Other asymptomatic reasons (*n* = 6)	35.17 (8.28)	44.17 (7.91)	8.83 (4.71)

Bootstrap Sampling Method = Simple, Bootstrap number of samples = 1000, Confidence Interval Type = Percentile, 95% confidence intervals

* *p* = 0.171, ¥ *p* = 0.539, β *p* = 0.709

*n* noun, *SD* standard deviation, *STAI-S* State Trait Anxiety Inventory - State, *STAI-T* State Trait Anxiety Inventory - Trait, *DAS* Dental Anxiety Scale, *TMD* temporomandibular disorder

### CBCT examination data

The κ coefficient was 0.89 for intraobserver agreement. MAs were observed in 6% of the patients’ images. Only one of the patients with MAs required re-imaging for reporting due to poor image quality.

Correlations between the presence of MAs and FOV, acquisition time, and anatomical area are given in Table 4. No statistically significant difference was found between the presence of MAs and FOV (chi-square, χ2 = 3.124, *p* = 0.673 > 0.05), acquisition time (Mann-Whitney U = 223, *p* = 0.400 > 0.05), or anatomical area (chi-square, Fisher’s *p* value = 0.597 > 0.05). The mean acquisition time was shorter in images with MAs than in those without and was in accordance with FOV size (not significant). MAs were not observed in images of the TMJ area.

**Table 4:** Presence of motion artefacts related to FOV, acquisition time, and anatomical area

MA	FOV* (cm)						Acquisition time^β^ (sec)		Anatomical area^π^	
	5 × 5	5 × 8	Double 5 × 5	Double 5 × 8	Triple 5 × 5	Triple 5 × 8	Mean	SD	Jaws	TMJ
+	(*n* = 2) 15.4%	(*n* = 1) 4.3%	(*n* = 0) 0.0%	(*n* = 2) 6.5%	(*n* = 0) 0%	(*n* = 1) 5.9%	20.274	10.055	(*n* = 6) 7.5%	(*n* = 0) 0%
-	(*n* = 11) 84.6%	(*n* = 22) 95.7%	(*n* = 4) 100%	(*n* = 29) 93.5%	(*n* = 12) 100%	(*n* = 16) 94.1%	23.739	9.963	(*n* = 74) 92.5%	(*n* = 20) 100%
	95% confidence intervals				Lower		−5.427			
					Upper		11.707			

Bootstrap Sampling Method = Simple, Bootstrap number of samples = 1000, 95% confidence intervals

* χ2 = 3.124, *p* = 0.673 > 0.05, ^β^Mann-Whitney U = 223, *p* = 0.392 > 0.05, ^π^ Fisher’s *p* value = 0.597 > 0.05

*MA* motion artefact, *+* present, − absent, *FOV* field of view, *Double* two horizontal stitched volumes, *Triple* three horizontal stitched volumes, *n* noun, *SD* standard deviation, *TMJ* temporomandibular joint

### Correlations between the presence of motion artefacts and patient characteristics

A statistically significant difference was found between the presence of MAs and patient age (*p* = 0.012 < 0.05, Mann-Whitney U). The mean age of the patients with MAs on their images (56.83) was higher than that for patients without (39.14). Of the 100 patients, 14 were over 60 years of age, and 86 were under 60 years old. Signs of MAs were observed in the images of two patients over 60 years old (14.28%) and four patients under 60 years old (4.65%).

Comparisons of MAs, anxiety scores and diagnostic reasons for CBCT imaging are given in Tables 5 and 6. There was no significant difference in gender (*p* > 0.05, chi-square), anxiety scores (*p* > 0.05, Mann-Whitney U) or diagnostic reasons for CBCT examination (χ2 = 6.404, *p* = 0.379 > 0.05) between patients with scans that showed MAs and patients with scans that did not. The frequency of MAs was higher in images of men (7.7%) than women (4.9%). The STAI scores of patients with scans that showed MAs were higher and the DAS scores lower compared with patients without MAs (Table 5).

**Table 5:** Mean STAI and DAS scores with standard deviation (SD) according to the presence or absence of motion artefacts

					95% confidence intervals for mean difference			
MA		N	Mean	SD	Lower	Upper	Mann-Whitney U	*P* ^a^
STAI-S	-	94	36.95	10.343	−11.981	3.375	205.000	0.184
	+	6	41.67	9.004				
STAI-T	-	94	41.43	8.765	−8.653	0.721	205.500	0.102
	+	6	45.00	5.441				
DAS	-	94	8.53	3.476	−2.679	3.636	249.000	0.999
	+	6	7.83	3.710				

^a^Bootstrap Sampling Method = Simple, Bootstrap number of samples = 1000, 95% confidence intervals

*MA* motion artefact, *N* noun, *SD* standard deviation, *P* significance level, *STAI-S* State Trait Anxiety Inventory - State, *STAI-T* State Trait Anxiety Inventory - Trait, *DAS* Dental Anxiety Scale, *+* present, − absent

**Table 6:** Comparison of motion artefacts and diagnostic reason for CBCT imaging

MA	Dental pain	Cyst/Tumour	TMD	Implant	Sinus pathologies	Trauma	Other asymptomatic reasons
+	6.8% (*n* = 3)	0.0% (*n* = 0)	0.0% (*n* = 0)	10.0% (*n* = 1)	0.0% (*n* = 0)	25.0% (*n* = 1)	16.7% (*n* = 1)
-	93.2% (*n* = 41)	100.0% (*n* = 12)	100% (*n* = 20)	90.0% (*n* = 9)	100.0% (*n* = 4)	75.0% (*n* = 3)	83.3% (*n* = 5)

*n* noun, *MA* motion artefact, *+* present, − absent, *TMD* temporomandibular disorder

## Discussion

The elimination of patient movement is important for obtaining sufficient image quality. The cause of MAs in CBCT images has been evaluated in several studies using different methods. Claustrophobia, being of old age or very young age, and fear of the CBCT procedure (related to movement of the C-arm) have all been suggested as reasons for patient movement during maxillofacial CBCT examination [4, 5, 10, 11, 29–32]. Patient anxiety is a possible reason for movement during CBCT scanning because anxiety has several emotional and physical symptoms, such as feelings of tension and jumpiness, restlessness, dizziness, shortness of breath, tremors and twitches. Research of the literature and systematic literature review studies [33] revealed that patient anxiety before maxillofacial CBCT scanning has never been evaluated and this is the first study to investigate the effect of patient anxiety on MAs in CBCT images.

The mean STAI-S score was 37.2 and the mean STAI-T score was 41.6 for the total population. Comparing the results of the present study to other investigations is not possible because studies assessing patient anxiety before CBCT examination are lacking. Previous studies have evaluated patient anxiety before several radiological modalities such as MRI and CT [14, 19–22, 34–36]. However, patients’ perceptions of medical imaging (such as diseases and scanning procedures) vary for dentomaxillofacial imaging, although there are some similarities, such as possible claustrophobic reactions and being worried about radiation exposure. The mean STAI scores of this study’s participants were lower than those reported in previous studies of patients without planned contrast medium applications using CT and MRI [22, 34, 37, 38]. This is because acceptance of those medical imaging methods is more difficult for patients. According to the mean anxiety levels, the population in the present study experienced anxiety before CBCT examination, with the mean STAI scores being higher than the normal scores of the population [18, 39]. The results of this study provide clues about how patients perceive CBCT.

This study was performed in a dentistry hospital, and dental anxiety was evaluated as a distinct variable, which differs from other previous medical imaging investigations. Patients usually experience anxiety before undergoing a dental procedure, and dental anxiety is a very common problem [40–42]. In this study, the increased STAI scores were in accordance with the rising DAS scores, indicating that dental fear provoked patient anxiety before CBCT examination.

Parallel to some previous studies that investigated patient anxiety before CT [22] and MRI scanning [43], no significant association was found between STAI score and patient age. However, DAS scores decreased with increasing age, which is consistent with earlier studies evaluating dental anxiety in different populations [44, 45]. Decreasing DAS scores in older individuals could result from adaptation to dental treatments over time [46]. Women demonstrated significantly higher STAI and DAS scores than men, and negative emotions such as stress, depression, fear, social phobia and panic have been reported as more frequent in females [47].

The prevalence of MAs in CBCT images was 6%. One patient with MAs required re-scanning to generate images of sufficient quality; in that case, CBCT was successful for diagnosis. Image quality is affected by the amount of movement—greater than 0.5 mm movement has the effect of destroying image quality [8, 48, 49]. In addition, MAs can be more visible in images with smaller voxel sizes [50]. Under these circumstances, minor patient movements might not have been recorded in this study. It is also possible to assess the patient motion from projected CBCT images by using optical flow measurements. However, it is difficult to detect small-vertical movements in the projection images [6]. In upcoming studies, applying a polyethylene tube or glass beads could be more effective for detecting smaller MAs.

According to the results of this study, FOV, acquisition time, and anatomical area are not related to MAs, consistent with earlier CBCT studies [4, 5]. However, this study showed that MAs are most frequently observed in images with shorter scanning times. In a previous study [10], patient movement was video-recorded during CBCT examination, and it was found that a majority of patients moved at the beginning of scanning. Based on these results, the authors concluded that patients do not tire during the scanning. The results of the current study are in accordance with these findings because if a patient did become tired during the scanning period, MAs would have been seen more frequently in images with a longer scanning time. Movement in the early phases of screening could be attributed to the rotation of the C-arm [10, 32] or to the patient following the device movement by turning his head. Scanning with the patient’s eyes closed is considered to reduce head movement [32], but it should be taken into account that anxiety complicates reflex control. Patients could also feel uncomfortable because of the noise and vibrations produced by the CBCT device [32]. These are triggering factors for anxiety. Demonstrating how the CBCT device works and showing the tube motion without radiation could be effective in decreasing reflexive motions and anxiety.

Guidance of a patient in a CBCT unit (standing/sitting/supine) and fixation of a patient’s head/chin can influence whether MAs occur [49]. The present study showed that anatomical area was not related to MAs; furthermore, MAs were not observed in the images of the TMJ area, which could be attributed to the difference in how the patients’ heads were supported in the CBCT unit, such as whether a cotton roll was used during the scan. More studies are needed regarding how guidance of the patient in the CBCT unit relates to MAs.

The results of this study showed that there was no relationship between patient anxiety and MAs; nevertheless, a non-significant increase in STAI score was noticed in patients with MAs on their images. Thus, there was a possible impact of patient anxiety on MAs in CBCT images. Regarding the diagnostic reasons for CBCT imaging, there were no significant correlations between anxiety level and the presence of MAs. Patient anxiety should be reduced before a CBCT examination through good communication that informs patients about the CBCT operation and by performing anxiety-reduction strategies with the patient. Further studies should focus on decreasing patient anxiety before CBCT imaging and measuring the impact of reduced patient anxiety on the prevalence of image MAs, in addition to investigating other possible factors that may provoke anxiety, such as fear of tube movement, claustrophobia, and fear of radiation.

The mean age of the patients with MAs on their images was significantly higher than that of patients without, which has also been found in other studies [4, 5, 11]. Patients over 60 years old are more likely to move due to the involuntary muscle movements that occur in this age group [1, 2, 4, 5, 11]. It is also well established that children under the age of 15 years are a risk group for patient movement during CBCT examination [4, 5, 10, 11]; however, only patients who were older than 18 years were included in the study. There is no department of orthodontics and paediatric dentistry at our hospital, so patients younger than 18 years are not referred for CBCT examination. Other limitations of this study include the relatively small sample size and the fact that data were collected from a single dentistry hospital. Despite the small number of participants, this study highlights that one should be aware of patient anxiety during CBCT imaging.

## Conclusions

Sample of patients experienced anxiety before CBCT scanning. Patients’ anxiety scores were significantly correlated with dental fear. Women showed higher anxiety levels than men. Excessive patient anxiety did not clearly affect image MAs during CBCT examination, although there was a non-significant increase in STAI scores for patients with MAs on their images. MAs were found to be related to increasing age.

## Abbreviations

CBCT: Cone beam computed tomography
CT: Computed tomography
DAS: Dental Anxiety Scale
FOV: Field of view
MAs: Motion artefacts
MRI: Magnetic resonance imaging
STAI: State Trait Anxiety Inventory
STAI-S: State Trait Anxiety Inventory - State
STAI-T: State Trait Anxiety Inventory - Trait
TMD: Temporomandibular disorder
TMJ: Temporomandibular joint
